# Deep‐Fat Frying of Chicken Nuggets: Impacts on Mass Transfer and Some Quality Indices

**DOI:** 10.1002/fsn3.70451

**Published:** 2025-06-22

**Authors:** Ipek Demirel Demiray, Haluk Ergezer, Engin Demiray, Özge Süfer

**Affiliations:** ^1^ Food Engineering Department, The Graduate School of Natural and Applied Sciences Pamukkale University Denizli Türkiye; ^2^ Food Engineering Department, Faculty of Engineering Pamukkale University Denizli Türkiye; ^3^ Food Engineering Department Osmaniye Korkut Ata University Osmaniye Türkiye

**Keywords:** chicken nugget, color, effective diffusion, frying, oil uptake, texture

## Abstract

This study examines the impact of deep‐fat frying on chicken nuggets' quality and mass transfer characteristics at three temperatures (160°C, 170°C, and 180°C) over 180 s. Moisture diffusion coefficients, calculated as 2.74 × 10^−8^, 8.22 × 10^−8^, and 13.69 × 10^−8^ m^2^/s, respectively, indicate increasing diffusion with temperature. The activation energy (*E*_*a*_) for moisture loss was found to be 128.81 kJ/mol. Initial moisture content was 0.0180 ± 0.003 kg water/kg dry matter, reducing by 28.33%, 31.11%, and 35% for each respective temperature, ending at 0.0129 ± 0.001, 0.0124 ± 0.002, and 0.0117 ± 0.002 kg water/kg dry matter. Oil uptake transfer coefficients were 0.0082, 0.0091, and 0.0094 s^−1^, with corresponding oil content values of 0.032 ± 0.003, 0.029 ± 0.003, and 0.026 ± 0.002 g oil/100 g dry matter. Equilibrium oil content decreased, while the *E*_*a*_ for oil uptake was −35.63 kJ/mol. Textural properties showed increases in hardness (129.88 ± 0.84 to 131.34 ± 0.56 mJ), springiness (4.28 ± 0.12 to 4.37 ± 0.12 mm), and chewiness (84.54 ± 0.56 to 85.65 ± 0.65 mJ) with temperature, while cohesiveness remained stable. Color measurements showed a brightness (L*) value decrease of up to 15.73% at 180°C and a total color change (Δ*E*) increase from 4.78 ± 0.55 at 160°C to 9.31 ± 0.76 at 180°C. This data provides a foundation for optimizing frying parameters to improve product quality and consistency in the food industry. Future research may explore the use of novel coating formulations or alternative frying media to minimize oil absorption while preserving desirable texture and flavor attributes.

## Graphical abstract

Higher frying temperatures reduced moisture content and oil uptake in nuggets. The moisture diffusion coefficient increased with temperature, aiding in water loss. Surface color darkened at higher temperatures, with total color change increasing. Optimizing frying parameters can improve nugget quality and reduce oil absorption.

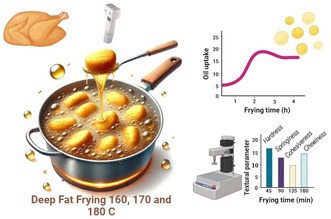

## 1 Introduction

The global demand for poultry meat continues to grow, because it is favored for lower fat content compared to red meats, high protein levels, and richness in essential vitamins and minerals (Kaymak Ertekin 2005; Connolly and Campbell 2023). To meet the growing demand for poultry, value‐added products like nuggets are being developed (Naghdi et al. 2025). Improving the quality of these products enhances the marketability of chicken. Items like schnitzel, nuggets, and breaded meatballs can be created by coating various poultry parts (such as thighs, breasts, wings, or meatballs) with different coatings and deep‐fat frying them (Kaymak Ertekin 2005). These coatings include liquid mixtures of water, flour, eggs, starch, and spices (Feng et al. 2022), while dry mixtures contain ingredients like flour, starch, and breadcrumbs, often used alongside the liquid mixtures (Kaymak Ertekin 2005).

The process of deep‐fat frying entails immersing food in oil heated to high temperatures (Juvvi et al. 2024), until the internal temperature reaches a safe minimum. Foods prepared through deep frying exhibit a hot and crispy exterior while ensuring thorough cooking at the center. This cooking method is characterized by its rapidity, and when executed correctly, it effectively eliminates bacteria. When the moisture present in food comes into contact with the extremely hot oil, it undergoes instantaneous vaporization, transforming into superheated steam. This steam expands rapidly, contributing to the formation of the characteristic crispy texture (Asokapandian et al. 2020). The process of deep‐fat frying is complicated because it requires the simultaneous transfer of mass and heat between the meal and the surrounding oil. The rate of heat transmission is greatly influenced by variables including specific heat, convective heat transfer coefficient, thermal conductivity, and thermal diffusivity (Sivaranjani et al. 2024). The main mass transfer mechanisms in deep‐fat frying are moisture loss and fat absorption, while the Fourier number for heat transmission falls between 0.1 and 0.5. Different food ingredients have mass transfer coefficients between 10^−4^ and 10^−6^ m/s and moisture diffusivity between 10^−6^ and 10^−10^ m^2^/s (Dash et al. 2022).

Processed chicken is used extensively in the fried chicken industry, one of the most well‐known segments of the food sector. The attractive flavor, appealing color, and crispy texture of fried products contribute significantly to their popularity. However, the frying process increases the overall fat content, leading to higher energy density. In response to growing consumer demand for healthier meat options, low‐fat meat products have been developed (Ananey‐Obiri et al. 2020; Das et al. 2013; Echarte et al. 2001; Sánchez‐Muniz and Bastida 2006). Gum‐based edible coatings have proven effective in reducing oil uptake during frying (Salehi 2020; Salehi et al. 2022). Moreover, combining edible coatings with sonication can further limit oil absorption, enhance the physical properties of fried products, and improve overall quality (Salehi et al. 2024, 2025).

Castro‐López et al.'s study (2023) examined the differences between deep‐fat frying and air frying for chicken nuggets at various temperatures. The activation energy for moisture diffusion was lower in air frying and greater in deep‐fat frying. Air‐frying at 170°C produced the greatest oil reduction, which was 25.6% less than deep‐fat frying. However, texture and color were impacted by air‐frying. Both approaches were used for different lengths of time. Besides that, air‐fried and deep‐fried chicken nuggets at the same temperature were tested by Cao et al. (2020), and deep‐frying increased the amount of moisture, oil, color, texture, volatile chemicals, and lipid breakdown. With less oil and fewer volatile components, air frying took longer to achieve comparable quality features. According to sensory evaluation, 9 min was the ideal frying time, and there was less oiliness and crispness. Moreover, research by Zhang et al. (2020) on battered and breaded fish nuggets highlighted the temperature‐dependent kinetics of fat absorption and moisture loss, which are central to achieving a consistent fried product. The kinetics of fat absorption and moisture loss were well represented by the first‐order kinetic model and Fick's second law of diffusion. With increasing frying temperatures, the transfer coefficients of these mass transports rose, suggesting that the microstructure of the crust is influenced by the temperature, which in turn impacts the kinetics of fat absorption and moisture loss.

This study focuses on analyzing key physicochemical properties (moisture and color) and textural attributes (hardness, springiness, cohesiveness, and chewiness) of chicken nuggets during deep‐fat frying at different temperatures. Additionally, kinetic parameters—including moisture diffusion, oil uptake transfer coefficient, and activation energy—were calculated to understand their influence on mass transfer during frying. In particular, a detailed kinetic analysis of both moisture diffusion and oil uptake was conducted at multiple temperatures. By simultaneously quantifying mass transfer coefficients, activation energies, and key quality parameters such as texture and color, this work offers a comprehensive basis for optimizing frying conditions. It was hypothesized that higher frying temperatures would enhance moisture loss, reduce oil absorption, and positively influence the texture and color characteristics of fried chicken nuggets.

## 2 Materials and Methods

### 2.1 Materials

Chicken breast meat and skins were purchased from a national poultry processing factory (Gedik Pilic Co., Uşak, Türkiye), along with commercial liquid and dry coating mixtures. They were brought to the laboratory at 4°C ± 1°C and minced through a 3 mm plate of a meat grinder (PM‐70, Mainca, Barcelona, Spain) to prepare the nuggets. Additional ingredients, including bread crumbs, black pepper, cumin, onion powder, salt, and sunflower oil for frying, were sourced locally.

### 2.2 Sample Preparations and Deep‐Fat Frying

In the study, doughs were prepared using 90% chicken mince, 5% bread crumbs, 3% onion powder, 1% salt, 0.5% black pepper, and 0.5% cumin (w/w). Stainless steel circle molds (5 cm diameter and 1 cm thickness) were utilized to shape the dough to approximately 30 g. The prepared nuggets were kept in the refrigerator (4°C ± 1°C) until coated. Refrigeration was done to increase the adhesion efficiency of the coating to the nuggets.

The chicken meatballs kept at refrigerator temperature were dipped for 10 s in a liquid coating solution prepared by stirring for 30 s in water at 45°C ± 1°C, then coated with dry coating and placed in the frying machine.

The nuggets were fried for 180 s at 160°C, 170°C, and 180°C. The oil temperature and the center temperature of the nugget (70°C ± 2°C) were measured with a thermoprobe during frying. In addition, at the end of each frying process, the oil level in the frying machine was checked, and oil was added if there was a shortage. The frying oil was replaced after a maximum usage time of 6 h. Color, moisture content, oil content, and texture were measured every 45 s to monitor quality changes during frying.

### 2.3 Moisture and Oil Analysis

Chicken nuggets were dried at 105°C in a forced convection oven (Memmert, Germany) until a consistent weight was reached in order to determine the moisture content. Following the drying procedure, the samples were left to cool in a desiccator, and the weight difference, which was stated on a dry basis, was used to determine the moisture content (Lalam et al. 2013).

Oil content was determined using the Soxhlet extraction method. To determine the oil content of fried nuggets, 100 mL of a methanol: chloroform (1:2) mixture was added to 10 g of the sample. This mixture was then homogenized in a blender (Waring, Germany). The shredded sample was filtered through filter paper in a separatory funnel. Since some more unfilterable residue remained on the filter paper during the filtration process, the sample was once again digested with 100 mL of methanol: chloroform solution and filtered again. The filtrates were combined, and 20 mL of 0.5% CaCI_2_ was added and shaken. The separatory funnel was then deaerated, and the sample was allowed to stand for 24 h for phase separation. At the end of this time, the lower phase was taken into cooled and tared oil flasks previously kept at 105°C for 2 h. The oil flask was distilled under vacuum at 40°C, and the flask that reached the desired dryness was weighed once again to determine the amount of oil content of the samples. The oil uptake (%) of the fried nuggets was calculated according to Equation (1) (Jouki and Khazaei 2022):

(1) $$\begin{array}{c} \text{Oil uptake}\;\left(\%\right)=\\ \frac{\text{Oil content of fried nuggets}-\text{Oil content of raw nuggets}}{\text{Oil content of}\;\mathrm{raw}\;\text{nuggets}} \end{array}$$

### 2.4 Kinetic Modeling of Mass Transfer

Moisture loss was modeled using Fick's second law of diffusion, providing a simplified view of moisture transfer in frying (Sosa‐Morales et al. 2006). In this study, it was assumed that internal resistance to moisture transfer was negligible and that the nugget samples could be approximated as infinite slabs, following the approach used in similar food systems. While this simplification allows for tractable modeling and analytical solutions, it is recognized that the multi‐layered structure of nuggets may introduce additional resistances not fully accounted for in this model. Given the relatively small thickness of the samples and the short frying times, this assumption was considered reasonable. The moisture diffusion equation is given in Equation (2).

(2) $$\frac{\partial M}{\partial t}=D\frac{\partial^{2}M}{\partial x^{2}}$$

where *t* is time (s), *D* is the effective diffusion coefficient (m^2^/s), *x* is the product's location coordinate (m), and *M* is the moisture content at a certain time (g/g, dry matter base). But according to Fick's second law, fried nuggets cannot be considered a distinct creature. This is because nuggets have two components: the crust and the center. Due to uneven initial moisture and post‐frying temperature distribution, Fick's law was applied only to the crust.

Nasiri et al. (2011) assumed a negligible initial homogeneous distribution of moisture and temperature in the crust of the nuggets, which can create resistance to mass transfer. Accordingly, an infinite slab is assumed, where mass transfer occurs from both sides of the specimens. The solution of the partial differential equation of Equation (2) is given in Equation (3).

(3) $$\mathrm{MR}=\frac{M-M_{e}}{M_{0}-M_{e}}=\frac{8}{\pi^{2}}\sum_{n=0}^{\infty }\frac{1}{{\left(2n+1\right)}^{2}}\exp\left(-\frac{{\left(2n+1\right)}^{2}\pi^{2}\mathrm{Dt}}{4L^{2}}\right)$$

where *L* is the sample's half thickness (m), *MR* is the moisture ratio, and *M*_*0*_ and *M*_*e*_ are the initial and equilibrium moisture contents (g/g, dry matter basis), respectively.

When the frying process reaches equilibrium, it is acceptable to infer that the moisture level is minimal, i.e., *M*_*e*_ = 0. To calculate the effective diffusion coefficient, Equation (3) was simplified and Equation (4) was obtained.

(4) $$\mathrm{MR}=\frac{M}{M_{0}}=\frac{8}{\pi^{2}}\exp\left(-\frac{\pi^{2}\mathrm{Dt}}{4L^{2}}\right)$$

A first‐order kinetic model given in Equation (5) was used to describe the oil uptake of the crust (Nasiri et al. 2011).

(5) $$\mathrm{FC}=O_{\mathrm{eq}}\left(1-e^{-\mathrm{kt}}\right)$$

where *FC* is the oil content at a specific time (g/g, dry matter basis), *O*_*eq*_ is the equilibrium oil content (g/g, dry matter basis), and *k* is the transfer coefficient of oil uptake (s^−1^).

Using an Arrhenius‐type equation found in Equations (6) and (7), the fluctuation of the equilibrium oil content (*O*_*eq*_) and effective diffusion coefficient (*D*_*eff*_) with temperature was ascertained (Nasiri et al. 2011).

(6) $$D_{\mathrm{eff}}=D_{0}\exp\left(\frac{-E_{a}}{\mathrm{RT}}\right)$$

(7) $$O_{\mathrm{eq}}=O_{0}\exp\left(\frac{-E_{a}}{\mathrm{RT}}\right)$$

where *T* is the absolute temperature in Kelvin, *R* is the universal gas constant (8.314 kJ/mol.K), *E*_*a*_ is the activation energy (kJ/mol), and *D*_*0*_ and *O*_*0*_ are frequency factors.

### 2.5 Texture Profile Analysis (TPA)

Five samples were taken from the fried nugget samples and measurements (hardness, springiness, cohesiveness and chewiness) were made with a texture analyzer (Brookfield CT3‐4500, USA) using a meat product probe from three different points on the front and back sides of each sample, and the averages of the measured values were taken (Dogan et al. 2005).

### 2.6 Color Analysis

The color values of the samples were determined at three different points during and after deep frying, keeping the conditions constant, using PCE‐CSM1 (PCE Instruments, UK) color meter (Ngadi et al. 2007). Total color change (Δ*E*) was derived from L*, a*, and b* values using Equation (8).

(8) $$∆E=\sqrt{{\left(L^{*}-{L_{0}}^{*}\right)}^{2}+{\left(a^{*}-{a_{0}}^{*}\right)}^{2}+{\left(b^{*}-{b_{0}}^{*}\right)}^{2}}$$

where ${L_{0}}^{*}$, ${a_{0}}^{*}$, and ${b_{0}}^{*}$ were measured from the non‐fried nugget sample.

### 2.7 Statistical Analysis

Every experiment was performed in triplicate. Statistical analyses were conducted utilizing Statistical Package for the Social Sciences (SPSS Version 18, IBM, USA) software. Before performing one‐way analysis of variance (ANOVA), the assumption of homogeneity of variances was evaluated. Subsequent to ANOVA, Duncan's multiple range test was employed at a 5% significance level to discern significant variations across means.

## 3 Results and Discussion

### 3.1 Moisture Loss and Oil Uptake

The initial moisture content of chicken nugget samples averaged 0.0180 ± 0.003 kg water/kg dry matter. Table 1 shows the moisture content values of chicken nugget samples at three different frying temperatures for 180 s, and no statistical differences in moisture content were observed from 90 s onward at 160°C (*p* > 0.05). A similar trend was observed in the moisture content values calculated from the 135th second of the frying process at 170°C (*p* > 0.05). At the end of all frying processes, there was a statistical difference between the moisture content values of the samples (*p* < 0.05). This stabilization in moisture loss aligns with findings by Teruel et al. (2015), where they observed that higher frying temperatures led to increased oil absorption and firmer texture due to moisture replacement by oil.

**TABLE 1:** Moisture content (kg water/kg dry matter) values of chicken nuggets.

Frying time (s)	160°C	170°C	180°C
0	0.0180 ± 0.003^aA^	0.0180 ± 0.003^aA^	0.0180 ± 0.003^aA^
45	0.0150 ± 0.002^bA^	0.0146 ± 0.001^bB^	0.0144 ± 0.002^bB^
90	0.0133 ± 0.002^cA^	0.0131 ± 0.003^cA^	0.0129 ± 0.001^cB^
135	0.0131 ± 0.001^cA^	0.0126 ± 0.002^dB^	0.0121 ± 0.002^dC^
180	0.0129 ± 0.002^cA^	0.0124 ± 0.001^dB^	0.0117 ± 0.002^eC^

*Note:* ^a–d^Means in the same row having a common letter are not significantly different (*p* < 0.05). ^A–C^Means in the same column having a common letter are not significantly different (*p* < 0.05).

As the frying temperature rose, the samples' moisture content dropped. As a matter of fact, the moisture content of chicken nuggets decreased from 0.0180 ± 0.003 to 0.0129 ± 0.001, 0.0124 ± 0.002, and 0.0117 ± 0.002 kg water/kg dry matter values in 160°C, 170°C, and 180°C for 180 s, respectively. While a 28.33% decrease occurred in the moisture content value of the samples fried at 160°C, a 31.11% and 35% decrease occurred in the moisture content of the samples at the end of the frying processes at 170°C and 180°C, respectively. The moisture content of deep‐fat fried chicken nuggets decreased similarly after 15 min at a temperature between 160°C and 190°C, according to Castro‐López et al. (2023). Adedeji et al. (2009) fried chicken nuggets at 170°C, 180°C, and 190°C in deep‐fat, after microwave pre‐cooking and without any pre‐treatment. A smaller decrease in moisture content in all samples fried at 170°C was found, while those fried at 190°C showed a greater decrease.

The oil uptake values of the chicken nugget samples were determined and given in Table 2. The oil uptake of the samples decreased with the increase in frying temperature. At the end of the frying process at 160°C, the oil uptake value of the specimens was 0.032 ± 0.003 g oil/100 g dry matter, while this value was 0.029 ± 0.003 at 170°C and 0.026 ± 0.002 g oil/100 g dry matter at 180°C. Statistically, there was no significant difference between the oil uptake values of the chicken nugget samples fried at 170°C and 180°C (*p* > 0.05). The oil uptake levels of chicken nuggets decreased over time during all frying processes.

**TABLE 2:** Oil uptakes (g oil/100 g dry matter) of chicken nugget samples in different conditions.

Frying time (s)	160°C	170°C	180°C
0	0	0	0
45	0.051 ± 0.002^aA^	0.038 ± 0.001^aB^	0.032 ± 0.002^aC^
90	0.039 ± 0.006^bA^	0.035 ± 0.002^bB^	0.029 ± 0.001^bC^
135	0.034 ± 0.004^bcA^	0.030 ± 0.002^cB^	0.028 ± 0.002^bB^
180	0.032 ± 0.003^cA^	0.029 ± 0.003^cB^	0.026 ± 0.002^bB^

*Note:* ^a–c^Means in the same row having a common letter are not significantly different (*p* < 0.05). ^A–C^Means in the same column having a common letter are not significantly different (*p* < 0.05).

The findings are currently in line with the literature. Nasiri et al. (2011) fried shrimp nuggets at three different temperatures (150°C, 170°C, and 190°C) in deep fat and reported that oil uptake decreased with increasing frying temperature. Higher frying temperatures often result in a better‐formed crust that operates physically and has less porosity, which reduces oil absorption owing to textural changes (Ran et al. 2023; Dana and Saguy 2006).

### 3.2 Modeling of Moisture Loss and Oil Uptake

There was a rapid decrease in the moisture level of samples at the beginning of the frying process at all temperatures, due to moisture loss on the surface (Adedeji et al. 2009; Nasiri et al. 2011; Ran et al. 2023). During frying, the water on the surface of the sample evaporates intensively, which leads to the formation of a crust, which in turn slows down the evaporation of moisture from the sample (Zhang et al. 2020).

Data on *D*_*eff*_ and *R* ^2^ values of chicken nugget samples, which were deep‐fat fried at specific temperatures, are given in Table 3. *D*_*eff*_ values obtained by frying chicken nugget samples at 160°C, 170°C, and 180°C were 2.74 × 10^−8^, 8.22 × 10^−8^, and 13.69 × 10^−8^ m^2^/s, respectively, and they increased with the increase in frying temperature. Because of the augmentation in process temperature, more moisture evaporated from the surface of the nuggets, and this situation triggered moisture transfer from the inner parts of the samples to the surface. Conversely, an Arrhenius‐type equation describes how temperature affects *D*_*eff*_. Plotting the *D*_*eff*_ values' natural logarithm versus the reciprocal of the absolute temperature (1/*T*) yielded a line (*m*) with a slope equal to (−*E*_*a*_/*R*). The *E*_*a*_ was found to be 128.81 kJ/mol using the ideal gas constant (*R* = 8.314 × 10^−3^ kJ/mol·K) and the computed slope value (*m* = −15,836).

**TABLE 3:** Effective diffusion coefficient values of chicken nugget samples with varying temperatures.

Frying temperature (°C)	*D*_*eff*_ × 10^−8^ (m^2^/s)	*R* ^2^
160	2.74	0.966
170	8.22	0.981
180	13.69	0.975

Castro‐López et al. (2023) calculated *D*_*eff*_ values as 1.46 × 10^−7^, 2.92 × 10^−7^, 4.28 × 10^−7^, and 5.19 × 10^−7^ m^2^/s for chicken nuggets deep‐fat fried at 160°C, 170°C, 180°C, and 190°C, respectively. On the other hand, the effect of temperature on *D*_*eff*_ is described by an Arrhenius‐type equation, and the *E*_*a*_ is calculated as 131.66 kJ/mol. Sobowale et al. (2019) optimized deep‐fat frying of goat meat sausage and calculated *E*_*a*_ values between 71.04 and 77.76 kJ/mol. Movahhed and Hossein (2019) estimated *E*_*a*_ values of deep‐fried hamburger slices with ultrasound pre‐treatment (40°C and 60°C) as between 3.20–17.29 kJ/mol in the boost mode of the process at 150°C, 160°C, and 170°C. In comparison, the *D*_*eff*_ values determined in our study were notably lower than those reported by Castro‐López et al. (2023) for deep‐fat fried chicken nuggets. Such differences may stem from variations in sample composition, coating characteristics, frying protocols, or initial moisture content. Furthermore, the E_a_ calculated in our work was slightly lower than the value reported by Castro‐López et al. (2023), yet substantially higher than those observed for goat meat sausages (Sobowale et al. 2019) and hamburger slices subjected to ultrasound pre‐treatment (Movahhed and Hossein 2019). These comparisons underscore the distinctive moisture transfer behavior and energy demands associated with the frying of coated meat products.

Fry oil can only enter areas where water evaporates, since oil pickup is mostly a surface process. Consequently, there is a connection between oil absorption and moisture loss (Mellema 2003; Durán et al. 2007). Generally, a greater moisture content should result in a lower oil content in the finished product (Mellema 2003; Nasiri et al. 2011). The frying phase and the chilling phase are the two separate stages of the oil absorption process in fried meals. These phases have different underlying processes for oil absorption. The cooling‐phase effect, surfactant hypothesis, and water–oil replacement are important processes that contribute to oil absorption. The cooling‐phase impact mostly occurs during the cooling step that follows frying, while water–oil replacement and surfactant theory primarily function during the frying phase (Xie et al. 2022). For all frying temperatures in this study, oil absorption rose with frying time, but it fell with rising frying temperature. These findings are in line with those of Adedeji et al. (2009) for chicken nuggets and Nasiri et al. (2011) for shrimp nuggets. On the other hand, frying temperature had no discernible impact on oil absorption, according to Miranda and Aguilera (2006). According to Troncoso and Pedreschi (2009) and Dana and Saguy (2006), oil uptake is generally reduced at higher frying temperatures for two main reasons: either a decrease in the porosity of the crust or the formation of a better‐developed crust that acts as a physical barrier between the food's interior and the surrounding oil.

Equation (5) was used to get the *k* values based on the oil content data, and Table 4 displays the associated parameter for the samples of deep‐fat fried chicken nuggets. Oil temperature, product type, pre‐treatment, frying circumstances, product width, and oil type are among the primary process factors that affect *k* values (Nasiri et al. 2011; Adedeji et al. 2009; Troncoso and Pedreschi 2009). Table 5 illustrates that, while *O*_*eq*_ reduced as frying temperature increased, the transfer coefficient value of oil absorption increased. This appears to support the idea that higher frying temperatures result in less oil absorption (Nasiri et al. 2011; Moyano and Pedreschi 2006).

**TABLE 4:** Transfer coefficients of oil uptake and equilibrium oil content values of chicken nugget specimens.

Frying temperature (°C)	*k* (s^−1^)	*R* ^*2*^	*O*_*eq*_ (g oil/g dry matter)
160	0.0082	0.958	0.1813
170	0.0091	0.951	0.1383
180	0.0094	0.958	0.1161

**TABLE 5:** Changes in color parameters during deep‐fat frying of chicken nuggets.

Color parameters	Frying temperature (°C)	Frying time (s)				
		0	45	90	135	180
L*	160	45.70 ± 0.35^aA^	47.43 ± 0.30^bA^	47.46 ± 0.21^bA^	44.72 ± 0.38^cA^	45.87 ± 0.29^aA^
	170	45.70 ± 0.35^aA^	44.42 ± 0.28^aA^	45.17 ± 0.07^aB^	42.54 ± 0.53^cB^	43.67 ± 0.19^dB^
	180	45.70 ± 0.35^aA^	46.33 ± 0.42^bC^	42.81 ± 0.45^cC^	39.47 ± 1.54^dC^	38.51 ± 1.42^dC^
a*	160	6.87 ± 0.33^aA^	5.35 ± 0.01^aA^	5.59 ± 0.16^aA^	6.72 ± 0.10^aA^	6.53 ± 0.33^aA^
	170	6.87 ± 0.33^aA^	7.39 ± 0.04^aA^	7.11 ± 0.13^aA^	7.77 ± 0.14^aA^	6.74 ± 0.27^aA^
	180	6.87 ± 0.33^aA^	5.17 ± 0.07^aA^	7.57 ± 0.21^aA^	6.69 ± 0.68^aA^	6.78 ± 0.28^aA^
b*	160	19.10 ± 0.45^aA^	18.40 ± 0.16^bA^	18.49 ± 0.20^bA^	19.24 ± 0.12^aA^	19.42 ± 0.40^aA^
	170	19.10 ± 0.45^aA^	20.48 ± 0.21^bB^	19.44 ± 0.31^aB^	17.69 ± 0.34^cB^	17.69 ± 0.31^cB^
	180	19.10 ± 0.45^aA^	17.45 ± 0.13^bC^	17.61 ± 0.12^bC^	15.74 ± 0.79^cC^	13.20 ± 0.93^dC^
Δ*E*	160	0	2.01 ± 0.32^aA^	2.62 ± 0.30^bA^	3.27 ± 0.25^cA^	4.78 ± 0.55^dA^
	170	0	2.34 ± 0.15^aA^	3.32 ± 0.39^bB^	4.19 ± 0.46^cB^	6.56 ± 0.38^dB^
	180	0	2.45 ± 0.44^aA^	3.71 ± 0.47^bB^	6.97 ± 0.38^cC^	9.31 ± 0.76^dC^

*Note:* ^a–d^Means in the same row having a common letter are not significantly different (*p* < 0.05). ^A–C^Means in the same column having a common letter are not significantly different (*p* < 0.05).

### 3.3 Effect of Frying Temperatures on Texture Characteristics

Texture is a crucial sensory characteristic that consumers use to evaluate food acceptability and make purchasing decisions. Traditional subjective sensory evaluations have limitations, but TPA offers an objective measurement methodology that correlates specific parameters with sensory textural attributes. This approach streamlines sample preparation and provides a valuable tool for quality control in food processing operations, making it a significant advancement in sensory analysis in both the food industry and scientific research (Rahman et al. 2021). Faloye et al. (2021) fried chicken nuggets at a temperature range of 155°C–175°C for 3–7 min by deep‐fat method. The researchers examined the changes in chewiness, springiness, hardness, adhesiveness, and cohesiveness values of the texture parameters of the samples. In another study, Tamsen et al. (2018) used different flours as coating materials in the production of chicken nuggets. The researchers then fried the nugget samples at 180°C for 30 s and examined some textural parameters.

**FIGURE 1:**
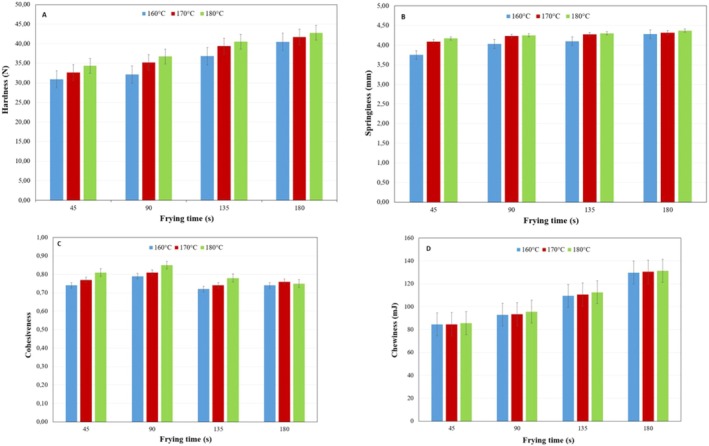
Variation of textural parameters of chicken nuggets. (A) Hardness, (B) springiness, (C) cohesiveness, and (D) chewiness.

Figure 1 illustrates that hardness, springiness, and chewiness increased significantly with temperature, while cohesiveness remained unaffected throughout the frying process. Hardness values of chicken nugget samples measured at the 45th, 90th, 135th, and 180th seconds at three different temperatures are given in Figure 1A. As the frying temperature rose, the samples' hardness levels increased as well. There was no statistical difference between the hardness values measured at 90th and 135th seconds of the frying processes at 170°C and 180°C (*p* > 0.05). Teruel et al. (2015) encountered a similar tendency when frying potatoes at different temperatures. The researchers explained this situation with the idea that the increase in temperature and time during deep‐fat frying may cause the food to take a hard structure by filling the gaps formed by the rapid evaporation of the water in the food by the oil.

Springiness values increased across all frying temperatures, with significant differences observed as early as 45 s (*p* < 0.05), indicating temperature's influence on structural integrity (Figure 1B). The springiness values increased during deep‐fat frying at three different temperatures. At the 45th second of frying at 160°C, 170°C, and 180°C, the springiness values were 3.75 ± 0.14, 4.09 ± 0.18, and 4.17 ± 0.12 mm, respectively. These values were found to be statistically different from each other (*p* < 0.05). The springiness values measured at 135th and 180th seconds of frying at 170°C and 180°C were not statistically different from each other (*p* > 0.05). Furthermore, the springiness values increased with the increase in frying temperature. At the end of frying at 160°C, 170°C, and 180°C, the springiness values of samples were determined as 4.28 ± 0.12, 4.32 ± 0.10, and 4.37 ± 0.12 mm, respectively (*p* < 0.05). Ran et al. (2023) fried fish balls with vegetables at 160°C for 360 s, examining certain quality characteristics at 60‐s intervals throughout the frying process (60, 120, 180, 240, 300, and 360 s). They reported that the springiness value, a texture parameter, was lowest at 60 s and highest at the end of the frying process (360 s). Although the numerical springiness value was greatest at 360 s, there was no statistically significant difference between the springiness values measured at 180, 240, 300, and 360 s (*p* > 0.05).

The cohesiveness values measured in chicken nugget samples during 180 s of frying at several temperatures are demonstrated in Figure 1C. There is no statistically significant difference between the cohesiveness values measured as a result of 180 s of frying at three different temperature levels (*p* > 0.05). In addition, the cohesiveness values remained consistent across temperatures and times (*p* > 0.05), aligning Şişik Oğraş and Kaplan (2022), who reported similar results across different frying methods (deep‐fat frying under atmospheric conditions and under pressure) and oil types (hazelnut and sunflower) for turkey nuggets. As a result of the study, using two different frying oils at 180°C under atmospheric conditions and at 160°C under pressure caused no statistically significant difference between the cohesiveness values of specimens (*p* > 0.05).

Like other textural parameters, the chewiness value was also specified in chicken nugget samples (Figure 1D). As in hardness and springiness values, chewiness values increased when the frying temperature increased. At 45 s of frying at 160°C, 170°C, and 180°C, the chewiness values were 84.54 ± 0.56, 84.66 ± 0.45, and 85.65 ± 0.65 mJ, respectively (*p* < 0.05). However, there was no statistically significant difference in chewiness values despite the increase in temperature across all frying times. For instance, chewiness values measured at 160°C, 170°C, and 180°C after 180 s of frying were 129.88 ± 0.84, 130.45 ± 0.63, and 131.34 ± 0.56 mJ, respectively, showing no statistically significant difference (*p* > 0.05). Unlike the current findings, Omidiran et al. (2022) observed a decrease in chewiness with rising temperatures in blue whiting ( *Micromesistius poutassou* ) nuggets, possibly due to variations in coating materials, nugget thickness, or frying time.

In order to better interpret the practical significance of the TPA results, it is important to consider how these instrumental parameters translate into consumer sensory perceptions. For example, increases in instrumental springiness generally correspond to a more elastic and resilient bite, which consumers perceive as a higher‐quality texture. Similarly, higher chewiness values reflect a denser and more cohesive structure, requiring greater mastication effort, and are often associated with a satisfying mouthfeel in fried products. Besides that, instrumental measurements of texture parameters often show good correlations with sensory perceptions such as firmness, elasticity, and mouthfeel, although these relationships are empirical and vary among products (Bourne 2002).

### 3.4 Effect of Frying Temperatures on Color

Color is arguably the most significant intrinsic sensory cue influencing our anticipations of the taste and flavor of food and beverages. Extensive laboratory research has established that modifications to the hue or intensity/saturation of various food and drink items can substantially alter consumer expectations, thereby affecting their subsequent sensory experiences. However, if the color does not align with the anticipated taste, it may lead to a negatively valenced disconfirmation of expectations (Spence 2024).

The results of the surface color parameters (L* a*, b*, and Δ*E*) of chicken nugget samples are depicted in Table 5. As frying temperature increased, L* values decreased, showing darker surface coloration, while a* values remained stable, and b* values showed a decrease at higher temperatures. L* serves as a crucial parameter within the food industry, as it is typically the primary quality attribute assessed by consumers in their evaluation of product acceptance (Salehi and Kashaninejad 2018). L* values of the samples decreased with the increase in frying temperature. Before frying, the L* value was determined as 45.70 ± 0.35. At the end of the frying process, the closest L* value to the initial form of the nugget (0th second) belonged to the samples fried at 160°C. There was a 15.73% decrease in the L* value of the chicken nugget fried at 180°C. There was a 4.44% decrease in the sample fried at 170°C (*p* < 0.05). Dogan et al. (2005) produced chicken nuggets with different coating agents and deep‐fat fried at 180°C for 12 min, and there was a decrease in L* values during and at the end of frying.

There was no clear decrease or increase trend in a* values of the samples fried at all temperatures during the process. For example, at 45 s of frying, a* values decreased at 160°C and 180°C, but increased at 170°C. A similar situation was encountered in the later stages of frying. There was no statistically significant difference between the a* values measured at the end of all frying processes and the initial a* values of the samples (*p* > 0.05). Also, there was no statistically significant difference between the a* values measured at the end of the frying processes at 160°C, 170°C, and 180°C (*p* > 0.05).

The b* value decreased at the end of deep frying at 170°C and 180°C, while it increased at the end of frying at 160°C. Generally, higher b* parameter values are associated with a greater yellow coloration in products, which is more advantageous (Salehi and Kashaninejad 2018). But this increase was not statistically significant (*p* > 0.05), and there was no difference between the b* value of initial (0th second) and last form (180th second) of the sample. There was a 7.38% decrease in the b* value of nugget samples fried at 170°C and a 30.89% decrease in those fried at 180°C.

Δ*E* values, representing the total color difference, increased notably with temperature, suggesting visible changes in nugget appearance, particularly at 180°C. Δ*E* values increased as the frying temperature increased (Table 5). At the end of frying at 160°C, 170°C, and 180°C, Δ*E* values were calculated as 4.78 ± 0.55, 6.56 ± 0.38, and 9.31 ± 0.76, respectively. It was also determined that Δ*E* values increased during the frying process at each temperature for 180 s (*p* < 0.05). The alterations in total color difference values can be attributed to several factors, including moisture loss, oil absorption by tissues, pigment degradation, and browning reactions. These changes are influenced by variables such as frying time, temperature, pressure, pretreatments, and pre‐drying processes (Manjunatha et al. 2019).

## 4 Conclusion and Future Remarks

This study explored the effects of deep‐fat frying on the quality and mass transfer characteristics of chicken nuggets at varying temperatures. The findings indicate that, higher frying temperatures led to a significant reduction in moisture content and oil uptake in the nuggets, demonstrating that elevated temperatures help to form a better‐developed crust, thereby reducing oil absorption. Specifically, temperature increases from 160°C to 180°C reduced oil uptake, providing a potential strategy for producing healthier products with lower fat content. Moreover, the diffusion coefficient for moisture increased with temperature, highlighting the efficiency of water loss at higher temperatures. This research contributes valuable data to optimize frying conditions for both quality preservation and consumer satisfaction, aligning with trends favoring healthier fried products.

Future studies could focus on alternative coating compositions or frying mediums to further reduce oil uptake without compromising texture and flavor. Additionally, incorporating techniques like vacuum frying or using ultrasound during the frying process could be explored to enhance crust formation while reducing oil absorption. Moreover, applying these findings to other food types and conducting sensory evaluations could provide broader industry applications. Nevertheless, the current modeling approach assumes negligible internal resistance and treats nuggets as infinite slabs, which represents a limitation. Future research could incorporate more advanced multi‐layer diffusion models to more accurately describe mass transfer behavior in structured foods, thereby advancing the understanding of frying dynamics and supporting the development of healthier, high‐quality fried products.

## Author Contributions

**Ipek Demirel Demiray:** formal analysis (equal), investigation (equal). **Haluk Ergezer:** conceptualization (equal), supervision (equal). **Engin Demiray:** data curation (equal), formal analysis (equal), investigation (equal), writing – original draft (equal). **Özge Süfer:** software (equal), visualization (equal), writing – original draft (equal), writing – review and editing (equal).

## Conflicts of Interest

The authors declare no conflicts of interest.

## Data Availability

The data that support the findings of this study are available from the corresponding author upon reasonable request.
